# Rhythmic qualities of jazz improvisation predict performer identity and style in source-separated audio recordings

**DOI:** 10.1098/rsos.240920

**Published:** 2024-11-13

**Authors:** Huw Cheston, Joshua L. Schlichting, Ian Cross, Peter M. C. Harrison

**Affiliations:** ^1^ Centre for Music and Science, University of Cambridge, 11 West Rd, Cambridge, UK; ^2^ Department of Psychology, Neuroscience & Behaviour, McMaster University, 1280 Main Street West, Hamilton, Ontario, Canada

**Keywords:** performer identification, music information retrieval, machine learning, stylometry, improvisation

## Abstract

Great musicians have a unique style and, with training, humans can learn to distinguish between these styles. What differences between performers enable us to make such judgements? We investigate this question by building a machine learning model that predicts performer identity from data extracted automatically from an audio recording. Such a model could be trained on all kinds of musical features, but here we focus specifically on rhythm, which (unlike harmony, melody and timbre) is relevant for any musical instrument. We demonstrate that a supervised learning model trained solely on rhythmic features extracted from 300 recordings of 10 jazz pianists correctly identified the performer in 59% of cases, six times better than chance. The most important features related to a performer’s ‘feel’ (ensemble synchronization) and ‘complexity’ (information density). Further analysis revealed two clusters of performers, with those in the same cluster sharing similar rhythmic traits, and that the rhythmic style of each musician changed relatively little over the duration of their career. Our findings highlight the possibility that artificial intelligence can perform performer identification tasks normally reserved for experts. Links to each recording and the corresponding predictions are available on an interactive map to support future work in stylometry.

## 1. Introduction

Great musicians have a style that is uniquely their own. In many cases, this distinctiveness can help explain why particular performers or composers have come to be regarded as ‘great’: typically, we tend to prefer music that is in some way novel and groundbreaking, over that which is overly familiar [1]. Learning to perceive the differences in style that are apparent between different musicians is a key aspect of learning to appreciate music and art in general. Perhaps for this reason, most modern curricula include components where students are required to listen to pieces of music and suggest possible performers or composers [2].

One style of music that is notable for its stylistic diversity is jazz, where performers make their own musical choices through improvisation, as opposed to these being predetermined by a composer. With sufficient training and experience, humans can learn to identify the improvisational styles of particular jazz musicians with remarkable accuracy. Paul Berliner has described this as a process by which aspiring musicians first come to discern ‘jazz licks’ from non-jazz melodic patterns, then ‘swing and bebop licks’, and finally ‘Charlie Parker and Sonny Rollins licks’ [3]. Taken to the extreme, in a famous ‘blindfold test’ published in the Downbeat magazine, trumpeter Miles Davis was able to identify the performers featured on eight different records by name, despite having never heard any of these recordings before [4].

Building computational models that can perform the same task is an important challenge facing Music Information Retrieval (MIR) research, as it allows us to decompose the expertise, knowledge and intuitions that listeners like Davis have built up over many years. In turn, this can help demystify exactly what differentiates one performer from another, with exciting potential implications: for instance, we can consider the relative importance of the different features that they have learned to use in making predictions, which allows us to interpret which aspects of music contribute the most towards defining individual performance ‘style’. These algorithms can also suggest possible performers for unattributed recordings, and as such invite comparisons with forensic authorship identification models previously developed in both linguistics [5] and visual art [6].

Previous MIR research in this area has mostly focused on identifying performers of ‘classical’ music, especially pianists. Here, as a ground truth exists in the form of a score, a performance can be evaluated in terms of deviations from what is expected given the notation, and a classifier can also be trained on several renditions of the same piece by different candidate performers. Both Saunders *et al*. [7] and Widmer and Zanon [8] used features relating to expressive timing and dynamics to identify the pianist in recordings of Mozart sonatas, with the latter also demonstrating a degree of transferability to music by other composers. Stamatatos and Widmer [9] expanded the feature set to include articulation and melody lead, all considered with relation to the score. More recent work has made use of advances in deep learning: Rafee *et al.* [10] employed hierarchical attention networks to model relationships between notes, beats, measures and phrases from automatically transcribed classical piano performances, with these again considered in terms of deviations from a ground truth, while Tang *et al.* [11] demonstrated that classification accuracy can be improved by training an artificial neural network on both original note-level data and score deviations.

Relatively little work has addressed performer identification in improvised music, where accurate notated transcriptions and scores are not available for the vast majority of performances and where a recording itself acts as a form of ‘ground truth’. Ramirez *et al*. [12] extracted phrase- and note-level data (including pitch, timing, amplitude and timbre) from recordings of different jazz saxophonists and evaluated several classification algorithms trained on these features, with ensemble learning methods performing the best. Edwards *et al*. [13] achieved impressive classification accuracy from using entire solo jazz piano improvisations (represented both with the original audio and transcribed ‘piano rolls’ containing timing, velocity and pitch information) as the input to an artificial neural network. However, this came at the expense of straightforward interpretations of the feature representations learned by the model. In addition, their audio-based model suffered from overfitting to the acoustic qualities of the recordings and the instruments they were made on, rather than learning to use cues that were musically informative and related to the qualities of the performer themselves.

Unlike much of the preceding work in this area, the focus of this article is not solely to advance the predictive accuracy of automatic performer identification models. Rather, we take an alternative approach, constraining the information provided to the model significantly in order to glean specific and interpretable insights about the diversity of musical styles in jazz and, especially, its focus on inter-performer interaction. We focus our attention solely on the rhythmic qualities of a jazz soloist’s improvisation style, which encompass both their anticipation and adaptation to their accompaniment, as well as the specific rhythmic patterns that they use. While a model trained on a more comprehensive suite of handcrafted features—or where feature representations are instead learned end-to-end from raw data—might achieve higher numerical accuracy, rhythm provides a consistent feature for analysing stylistic expression across the genre and, unlike pitch, harmony or timbre, is relevant to any instrument.

Previous research has also indicated that jazz performers display substantial differences in their use of rhythm, which makes this feature ideal for training a classification model. These differences between performers encompass variability in ‘swing’—the characteristic subdivision in jazz of the musical pulse into alternating long and short intervals [14–16]—and ‘feel’—the temporal relationship and synchronization between their and others’ performance in an ensemble, commonly referred to as playing either ‘ahead’ of or ‘behind’ the beat [17–19]. Evidence from other forms of improvised music also suggests that performers may vary in terms of their ‘complexity’—the amount of distinct rhythmic information they impart [20]—and ‘interaction’—the degree to which they adapt to match any temporal variation in the performances of the other musicians in their ensemble [21].

We designed an automated pipeline that enabled rhythmic features relating to these categories to be extracted from commercial recordings of jazz piano trios, an ensemble that consists of a piano soloist improvising with bass and drums accompaniment. First, we split a trio recording into three separate audio signals (one for each instrument) by applying an audio source separation model. We extract event onset data from each individual ‘stem’ using signal processing and derive our features from quantitative analysis of these data. Our approach takes advantage of recent developments in source separation that have made it practical to apply automatic transcription algorithms to commercial audio recordings, where the individual performances of multiple musicians are combined into one audio signal. While we used our pipeline to study jazz piano trios due to the wealth of models capable of separating these instruments from an audio signal, our approach could eventually be extended to other ensembles across a variety of genres.

Our dataset consists of rhythmic features extracted automatically from 300 commercial recordings by 10 different jazz pianists, totalling approximately 12 hours of audio data. These musicians were identified from listening and discographic data scraped from several Internet databases and are among the most popular and prolific pianists to have been active in the trio format. The selected recordings all exhibited characteristics of a traditional, mainstream style of jazz improvisation (commonly referred to as ‘straight ahead’), ensuring a relatively consistent set of tracks for analysis. The rhythmic features we extracted were selected both due to their prevalence in the existing quantitative literature on jazz timing, as well as a sense arising from prior qualitative and ethnographic work that jazz performers commonly used these terms when evaluating their and others’ improvisation styles [3,22]. We have released this dataset under a permissive, open-access license to facilitate further research [23].

In the methodology section, we further describe our dataset, as well as the construction of the feature extraction pipeline. The results section evaluates the efficacy of these features when predicting the improvisational style of individual pianists and explores their utility in identifying distinct stylistic clusters. Finally, we describe the study limitations and propose potential directions for future work.

## 2. Material and methods

### 2.1. Dataset construction

Our dataset is Jazz Trio Database (JTD)—described in detail in [23]. JTD contains annotations of 1294 piano trio recordings made between 1947 and 2015 and features 238 unique musicians. JTD was created by first scraping the top 250 000 artists or groups most frequently tagged with the genre ‘Jazz’ by users of the Last.fm platform, removing artists who did not appear in one of two well-known jazz discographies and scraping the MusicBrainz metadata database to identify artists with at least 1 hour of recordings in the piano, bass and drums trio format. Recordings that did not meet a defined inclusion criterion, including an average tempo between 100 and 300 quarter note beats-per-minute (BPM), a time signature of either three or four quarter note beats-per-measure and an uninterrupted ‘swing eighths’ feel, were then removed, with the goal of ensuring that JTD reflected the ‘straight ahead’ form of jazz as practised over the last century.

**Figure 1.**
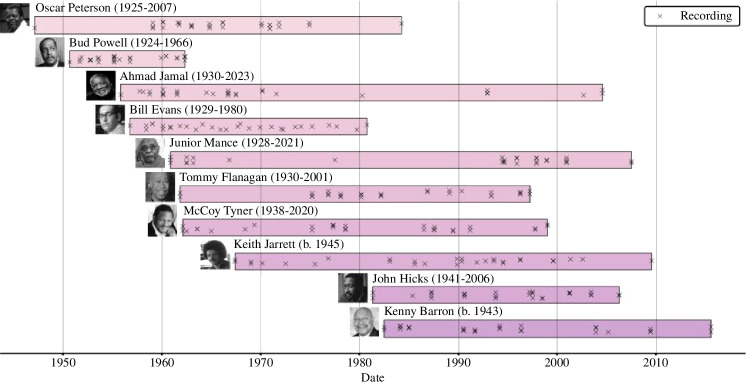
Corpus construction. Each horizontal bar shows the duration of the recording career for each of the 10 pianists considered here; markers indicate the date of recordings sampled in JTD-300, randomly jittered horizontally and vertically for visual clarity.

In this work, we use a balanced subset of the full database called JTD-300 (electronic supplementary material, figure S1), which consists of 30 improvised solos each by the 10 pianists with the most amount of material in the full JTD. The duration of recordings by these musicians constituted 26.4% of the duration of the entire JTD, and thus, they can be considered prolific exponents of the trio format. To create JTD-300, individual recordings were sampled as evenly as possible from each pianist’s discography. Tracks were arranged chronologically by recording date and original track listing, sorted into 1 of 30 equally spaced bins (starting the day of their earliest recording and ending the day of either their most recent or final recording), and sampled sequentially from each bin until 30 recordings were obtained for every pianist (figure 1).

Only audio corresponding to the piano solo in each recording was used, as the improvised music in the solo originates from the performers rather than from a composer. Together, the solo excerpts amounted to 11.75 hours of audio, with an average duration per excerpt of 2 minutes, 21 seconds. Each recording was split into three separate audio signals, corresponding to every instrument in the trio, using the *ZFTurbo* source separation model [24]. Additional post-processing was applied to the piano source, consisting of a second-order Butterworth bandpass filter set to attenuate frequencies lying outside the range of 50−3520 Hz (encompassing the fundamental frequencies of the pitches *A*_1_–*A*_7_ on a piano tuned at *A*_4_ = 440 Hz). This helped reduce any residual ‘spill’ between instruments and led to better results than annotating onsets in the raw audio signal [23].

Onsets were detected in the piano, bass and drum audio signals using the convolutional neural network processor contained in the madmom Python library (v. 0.16.1) [25]. Quarter note beat and downbeat positions were estimated by applying both the recurrent neural network and dynamic Bayesian network processors from the same library. These beat trackers were applied three times to the raw audio mixture, with the permissible duration of detected inter-beat intervals gradually narrowing each time according to the distribution of the returned values; the output of the third pass of the network was used to derive the beat positions. This process was found to improve performance over applying the beat trackers only once [23]. The default setting of 100 frames per second was used for both beat tracking and onset detection, such that near-simultaneous events with a time difference of less than one frame (likely including, e.g. piano chords) would be treated as a single onset.

To validate the detection pipeline, ground truth annotations were created by human annotators for 34 tracks; the earliest, middle and final recording in JTD-300 by each pianist, alongside four additional recordings by Bill Evans (the pianist with the most audio in JTD). The gradient-free, nonlinear optimization algorithm *subplex* [26] was then applied to fine-tune the parameters used to pick peaks from the activation functions returned by the neural networks, with the mean *F*-measure treated as the objective function to maximize. The implementation of this algorithm was taken from the *nlopt* Python library (v. 2.8.0) [27]. The window used to match ground truth and automatic annotations was set to ±50 ms. After optimization, a mean *F*-measure of 0.93 (SD = 0.03) was obtained for piano onset detection and 0.97 (SD = 0.05) for beat detection. This indicated that the pipeline was producing broadly accurate results for both onset and beat classes. The final parameter set used for piano onset detection is given in electronic supplementary material, table S1.

Each beat tracker timestamp was matched with the nearest onset played by every musician to estimate where they marked the underlying pulse, using a window of one 32nd note before the beat and one 16th note after, according to the estimated tempo. Our rationale for using an asymmetric window came from previous research which has suggested that jazz soloists are more likely to ‘lag behind’ and mark the beat later than their rhythm section, as opposed to earlier [17,19], and that notes played more than a 32nd note earlier than a given beat have been considered a subdivision of the previous beat (i.e. a delayed, swung ‘eighth’ note), as opposed to marking that beat itself [16]. If no onsets were included within a window, then the musician was considered to not have marked the pulse at that beat.

### 2.2. Feature extraction

**Figure 2.**
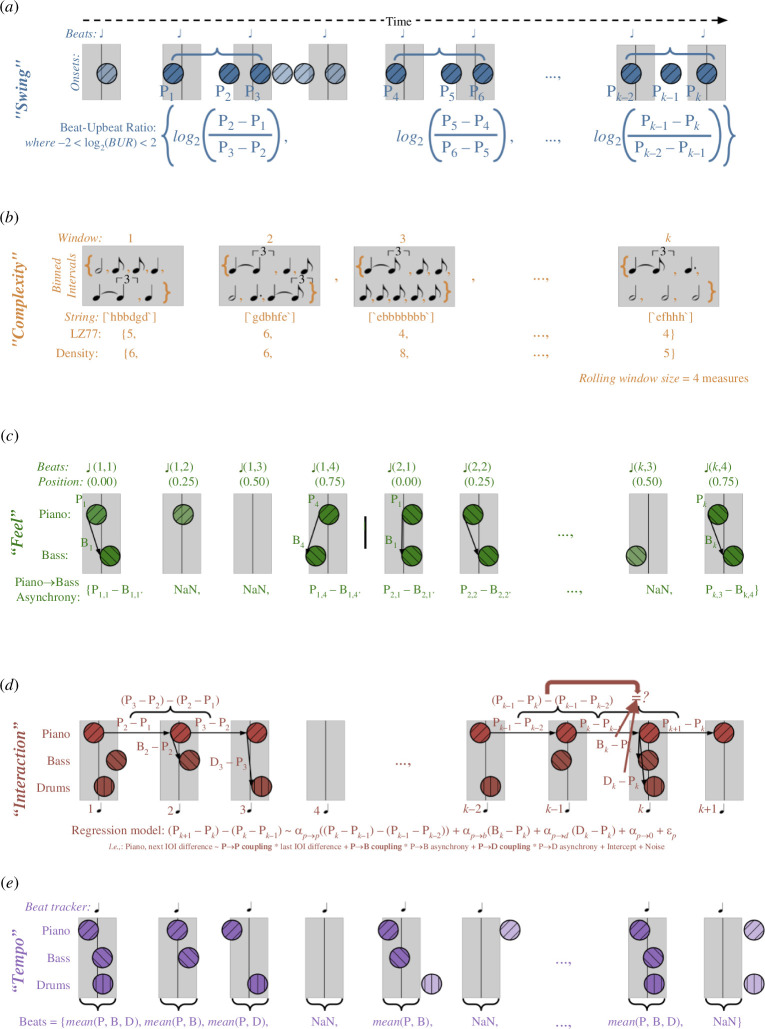
Feature extraction process. Each panel shows the procedure used to extract features relating to one of five categories: (*a*) ‘swing’, (*b*) ‘complexity’, (*c*) ‘feel’ (piano→bass only), (*d*) ‘interaction’ and (*e*) ‘tempo’. In all but (*b*), coloured circles represent onsets and grey rectangles the window around which these were matched to tracked beats (see Dataset Construction, above). In (*b*), grey rectangles represent the sliding four-measure window to which the LZ77 algorithm was applied. Time progresses from left to right in every panel.

Data extracted from JTD-300 consisted of 236 228 piano onsets, 98 159 of which were matched with a quarter note timestamp from the beat tracker (41.6%). We adopted a bag-of-features approach to represent each track in JTD-300 using 19 individual features; these low-level features were grouped together into five higher-level categories (‘swing and snap’, ‘complexity’, ‘feel’, ‘interaction’, and ‘tempo’). In figure 2, we provide diagrams showing how these features were extracted from the onset data.

#### 2.2.1. ‘Swing’ and ‘Snap’

The concept of swing is central to jazz performance practice. While it has aesthetic connotations (‘the band was swinging’), the technical usage of the term refers to the uneven subdivision of the quarter note into both a long and short interval. In Western musical notation, the long interval is typically understood as a quarter note triplet, and the short as an eighth note triplet; this has led to the shorthand expression ‘swung eighths’, which is often notated for convenience as straight eighth notes (with the swing taken as implied).

Swing can also be expressed numerically in terms of the ratio between the duration of the long and short intervals—known as the beat-upbeat ratio [15]—with notated swing eighths equivalent to a 2 : 1 ratio (figure 2*a*). Rather than following this pattern exactly, previous research has shown that swing as it is performed is often closer to a 1 : 1 ratio, equivalent to the equal subdivision of the quarter note. Put differently, when compared with the expected prototype, the long interval is shorter and the short interval is longer [16].

Levels of swing can vary between different musicians, even those who play the same instrument [14–16,18,28]. However, the degree to which variation in swing is truly indicative of stylistic diversity between performers is unclear, given that their swing phrasing can also vary as a function of tempo, ensemble role and rhythmic ‘feel’ [14,16,23,28]. We investigated this issue by searching JTD-300 for all discrete groupings of three onsets where the first and last onset both marked the quarter note pulse, i.e. a quarter beat interval with one subdivision. From these groupings, we extracted the beat-upbeat ratio, expressed in binary logarithmic form. The total number of such groupings in JTD-300 was 41 815. Following the analysis of the Weimar Jazz Database in [16], we classified beat-upbeat ratios above 4 : 1 (log_2_ = 2) and below 1 : 4 (log_2_ = −2) as outliers. This resulted in the loss of 493 (1.2%) values.

As a result, our analysis includes both short-long and long-short subdivisions of the quarter note; the former have a beat-upbeat ratio below 1 : 1 (log_2_ < 0.0) and are defined as ‘snap triples’ in [16]. While these rhythms may be used intentionally by musicians, they appear more frequently at faster tempi [14,16,23], where they could instead result from inaccuracies in maintaining either the long-short or even-even pattern at high speed. Excluding these values from the analysis could potentially introduce bias into the model when a given performer predominantly played faster or slower pieces. Including short-long subdivisions of the quarter note in analysis of the beat-upbeat ratio is also consistent with previous work on jazz timing [14–16,23]. The mean log_2_ beat-upbeat ratio obtained for recordings in JTD-300 was 0.34 (SD = 0.59), with 13.2% of these being ‘snap’ triples (i.e. log_2_ < 0.0).

#### 2.2.2. Complexity

In information theory, the complexity of a variable or process refers to the minimum amount of information that is required to represent it. Estimators of complexity typically reflect the randomness, unpredictability and intricacy of a sequence, including the difficulty involved in predicting an upcoming value from all previous ones [29]. The presence of repeated patterns, for instance, lowers the complexity of a sequence as future portions can be reconstructed from earlier ones, allowing for a more concise and compressed description of the total [30]. In music research, rhythmic complexity has been understood to relate to both the predictive processes that occur during listening, as well as the pleasurable urge to move to music [1,31,32].

We assessed information-theoretic complexity using the ‘LZ77’ compression algorithm [33], which identifies patterns in sequences by sliding a buffer over the input data and searching for repetitive occurrences within that buffer. Sequences achieve low complexity if they contain segments that can be represented by referencing previously encountered portions. In the context of a musical performance, this algorithm can be applied to analyse the temporal relationships between inter-onset intervals and identify recurrent rhythmic patterns, enabling the perceived complexity of a sequence to be assessed based on the variation, density and arrangement of rhythmic durations [34]. This algorithm has previously been used in MIR research for tasks including cover song identification [35], structure modelling [36] and music analysis [37].

Standard information-theoretic measures of complexity such as LZ77 are only defined for discrete random variables that can take on a countable number of distinct values, like the number of heads in a series of coin tosses. Due to the presence of microtiming, sequences of inter-onset intervals instead follow a continuous distribution and have to be converted to a discrete sequence before their complexity can be calculated using these methods.

One way this can be accomplished is by grouping continuous data into bins. We chose to bin every inter-onset interval in JTD-300 according to its closest approximate rhythmic value within the duration of a full measure. Ordered from shortest to longest notated rhythm, the six bins used corresponded to the (1) triple and (2) duple subdivision of the quarter note, the (3) quarter note itself, the (4) triple and (5) duple subdivision plus a quarter note and (6) the half note (see notation in electronic supplementary material, figure S2). The edges of each bin extended to midway between those on either side; in the case of the shortest (1) and longest rhythmic value (6), either zero or the full duration of a measure was used instead. Values lying on the boundary between two bins were grouped into whichever had the longer duration and values that could not be grouped into a bin were classed as outliers.

Our assumption was that rhythmic complexity would change over time within a performance and would be best assessed over short time spans, rather than complete performances. Therefore, we measured complexity across a sliding window of four measures in duration—this being a standard length for a melodic phrase in jazz—with three measures overlap (i.e. a distance of one measure separated two successive windows). The LZ77 algorithm was then applied to string representations of the binned inter-onset intervals contained within each window (figure 2*b*). We obtained 33 865 such compression scores across JTD-300, with a mean compression score of 11.1 (SD = 1.9). Note that the binned inter-onset intervals were only used in the calculation of features deriving from the LZ77 algorithm; all other features made use of ‘raw’ inter-onset interval values, with microtiming preserved.

We also extracted the density of each sliding window (i.e. the number of inter-onset intervals it contained), which was positively correlated with the measured complexity of the window; across individual recordings in JTD−300, mean *r* = 0.41 (SD = 0.25). The average density of a window of four measures was 26.1 onsets (SD = 8.6)—meaning that, assuming four beats-per-measure, there were approximately 1.6 piano onsets per quarter note. Note that this is not the same as 1.6 notes per beat; for instance, chords are classified as a single onset, with the parameters of the onset detection algorithm (including the number of frames processed per second) affecting the degree with which perceptually simultaneous events are treated as unique onsets.

#### 2.2.3. Feel

In an improvised performance—and, indeed, in classical music [38]—just as important as the notes a musician plays is when they choose to play them. The flow of musical time is flexible in jazz, and there are subtle nuances in how different performers choose to articulate the pulse. These subtleties are often understood as a performer’s rhythmic ‘feel’, which can involve purposely playing before or after the expected position of the beat—variously referred to as pushing, pulling, rushing or dragging [3,19,22,39]. Expert musicians can vary their feel depending on who they are playing with and what they are playing: certain pianists may prefer accompanists who articulate the beat in specific ways [3], while particular styles might lend themselves to different feels [40].

We calculated a pianist’s rhythmic ‘feel’ as the temporal difference between their onsets that were matched with the quarter note beat, and the equivalent onsets matched to the same beat that were played by the drummer and bassist. While this could have been expressed in ‘raw’ units (e.g. milliseconds), given the range of tempi in JTD-300, we instead expressed it as a percentage of the duration of a quarter note at the estimated tempo of a track. A value of +25% would thus imply that one musician played a 16th note later than another (figure 2*c*). Averaged across JTD-300, pianists played 4.5% (SD = 9.7) the duration of a quarter note after bassists and 6.0% (SD = 9.3) after drummers.

#### 2.2.4. Interaction

While the analysis of rhythmic feel is interesting insofar as it can reveal where the pianist marked the pulse, it cannot illuminate how the rest of their ensemble might have reacted to this. A group of musicians remains synchronized through constant adaptation to temporal variation in each other’s performances. When one performer adapts to match discrepancies in another’s timing, we can say that they are influenced by—or ‘coupled’ to—them; vice versa, the absence of adaptation indicates that they are not influenced by that performer [41].

There are numerous methods described in the literature that are suitable for modelling the reciprocal adaptation and coupling within a musical ensemble: for a recent review, see [42]. One of the best established is the linear phase correction model implemented in [43], where the duration of a performer’s upcoming inter-beat interval is predicted from both the duration of their prior inter-beat interval and the asynchrony with their partner(s) at the previous beat. In other words, in this model, higher levels of coupling imply a greater degree of temporal correction and anticipation to a partner than would be present at lower levels of coupling [21,41,43].

In an earlier study, we demonstrated good results from using linear phase correction to model the interaction between a jazz pianist and drummer in an experiment [44], which led us to apply the same model to commercial recordings here. As in this previous study, to control for any global drift in performance tempo we expressed every quarter note inter-beat interval inputted into the model in terms of its difference from the preceding interval (figure 2*d*).

We applied the phase correction model separately to the performance of the pianist, bassist and drummer in every recording; thus, with 300 recordings in the database, *n* = 900 models. By doing so, we were able to model adaptation both ‘to’ and ‘from’ the piano soloist. As the robustness of this model varies depending on the amount of data given to it, in cases where fewer than 30 observations could be obtained for a given performer in a given track (i.e. the number of predictors in the model, multiplied by 10), it was classified as an outlier. This resulted in the exclusion of 55 models (6.1%). Across the remaining models, mean *R*^2^_adj_ = 0.61 (SD = 0.13), which suggested that, on average, linear phase correction captured slightly less than two-thirds of the variation in differenced inter-beat intervals.

The mean influence of the piano soloist on the bassist was 0.15 (SD = 0.13) and on the drummer was 0.14 (SD = 0.12). Vice versa, the mean influence of the bassist on the piano soloist was 0.40 (SD = 0.28) and of the drummer was 0.59 (SD = 0.32). These coefficients can be interpreted as the predicted change (in seconds) between the pianist’s upcoming and previous inter-beat interval, for every 1 second increase in asynchrony with the coupled instrument; thus, larger values imply that a greater degree of correction to any previous asynchrony took place on the immediately following beats.

Finally, the mean pianist self-coupling, equivalent to the influence of their own prior beat durations on future durations, was −0.52 (SD = 0.09). In total, these values are all within the range expected for stable performance by a small ensemble [43] and which have been demonstrated in previous studies of improvisation within both jazz rhythm sections [44] and Malian drum ensembles [21]. For recordings in JTD-300 where a model was excluded due to a lack of observations, missing coefficients were replaced using these global means.

#### 2.2.5. Tempo

We extracted four additional features relating to the tempo of the performance (figure 2*e*). These features were the performance tempo, equivalent to the mean of all inter-beat intervals; the performance tempo slope (specifically, the signed overall tempo change per second in a recording), equivalent to the slope of a linear regression of instantaneous tempo against beat onset time, such that positive values imply net acceleration and negative deceleration [44]; the tempo stability, calculated by taking the standard deviation of the pianist’s inter-beat intervals along a sliding four-measure window, then calculating the median of all windows [44]; and the percentage of tracked quarter note beats that could not be matched with a piano onset, reflecting whether the performer favoured playing ‘off’ or ‘on’ the beat.

Both the tempo stability and unmatched beats features used beat-matched onsets extracted from the pianist’s performance. However, the performance tempo and tempo slope features used beat-matched onsets obtained from every musician in the trio, rather than just the pianist. This was to reflect the fact that, in this style of jazz, musicians are expected to play together—maintaining the same global tempo and slowing down or speeding up with each other. These two features used the average of all onsets matched with a single-beat tracker timestamp for each musician in the trio, provided that onsets from at least two musicians were matched in the first place. This was true for 89.5% of beat tracker timestamps, with all other timestamps set to missing and excluded from the feature extraction process.

The mean values extracted for each feature were: 195.00 (SD = 50.02) BPM for performance tempo; +0.02 (SD = 0.04) beats-per-minute-per-second for tempo slope; 26.17 (SD = 10.35) ms for tempo stability (equivalent to 9% the duration of a quarter note at the average performance tempo) and 28.06% (SD = 8.80) for the unmatched beats feature.

#### 2.2.6. Feature set

In table 1, we summarize both the high-level categories and low-level features extracted from every recording, as described above. Note that, in cases where multiple values of a single feature were aggregated across a performance (e.g. LZ77 compression scores, beat-upbeat ratios), the standard deviation was included as an input to the model as well as the mean, so as to capture the variability of this feature within the solo.

**Table 1.** Bag-of-features. A summary of the high-level categories and low-level individual features used in the model.

category	number	feature description
‘swing and snap’: the ratios between the duration of ‘eighth note’ rhythms within the span of one beat.	1	mean, log_2_ beat-upbeat ratios
	2	standard deviation, beat-upbeat ratios
‘complexity’: the density and unpredictability (measured as compression rate) of the rhythmic information conveyed in groupings of four measures.	3	mean compression (LZ77) scores, across all groupings
	4	standard deviation compression scores
	5	mean onset density
	6	standard deviation onset density
‘feel’: the relative position of the pianist compared to the bass and drums, expressed relative to the tempo and meter of the performance.	7	mean piano-bass asynchrony
	8	standard deviation piano-bass asynchrony
	9	mean piano-drum asynchrony
	10	standard deviation piano-drum asynchrony
‘interaction’: the pianist’s adaptation to temporal variation in the bass and drum performances (and vice versa), modelled using linear phase correction.	11	pianist modelled coupling to bassist
	12	pianist coupling to drummer
	13	drummer coupling to pianist
	14	bassist coupling to pianist
	15	pianist self-coupling
‘tempo’: calculated according to onsets matched with the beat tracker timestamps.	16	performance mean tempo
	17	performance tempo slope
	18	pianist tempo stability
	19	fraction of tracked beats without a matching onset

**Figure 3.**
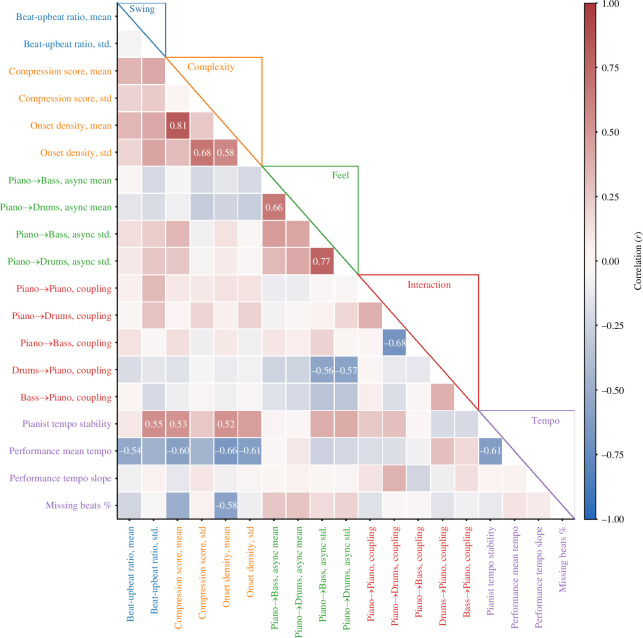
Pairwise correlations between features. The strength of the correlation (Pearson’s *r*) between two features is shown by the colour of the cell at the intersection of x- and y-axis variables: darker colours indicate stronger correlations. Text annotations are provided only where *r* > 0.5.

Figure 3 shows the correlation between pairwise combinations of these features. In particular, we noted that performance tempo correlated negatively with both mean beat-upbeat ratio, compression score, onset density and overall tempo stability, insofar as faster performances swung less, featured fewer onsets and less complex rhythms in an average group of four measures, and were more stable overall; piano–bass coupling correlated negatively with piano–drum coupling, suggesting that pianists followed either one or the other accompanying instrument, but not both; and drum–piano coupling correlated negatively with the standard deviation of piano–bass and piano–drum asynchronies, meaning that pianists who demonstrated greater variability in their rhythmic feel also had less influence on drummers.

## 3. Results

### 3.1. Rhythmic features predict jazz performer identity

We fitted a random forest classification model to predict the identity of the pianist in a recording from JTD-300 using our bag of 19 features. The implementation was taken from the *scikit-learn* Python library (v. 1.3.0) [45]. The random forest classifier is a supervised machine-learning algorithm that fits multiple decision trees to bootstrapped subsamples of data, with each tree ‘voting’ on a given class and the majority vote taken as the predicted label of the whole ensemble. The random forest is robust both to a large number of predictors and to nonlinear relationships between them and has been used widely in prior work on stylometry and authorship attribution [6,12].

Following a randomized search over a two-dimensional parameter space (*n* = 10 000 iterations), a forest size of 114 decision trees (each with a maximum depth of 86 nodes) achieved ceiling accuracy. Splits within every individual tree were performed considering a randomized subsample of floor (log_2_f) total features, where f was the total number of features in the bag. Thus, the values obtained from four individual features, sampled at random, were considered at each split. This was also found to be optimal during the hyperparameter search, compared with either *f* or floor ($\sqrt{f}$) total features. We show the full parameter settings after optimization in electronic supplementary material, table S2.

**Figure 4.**
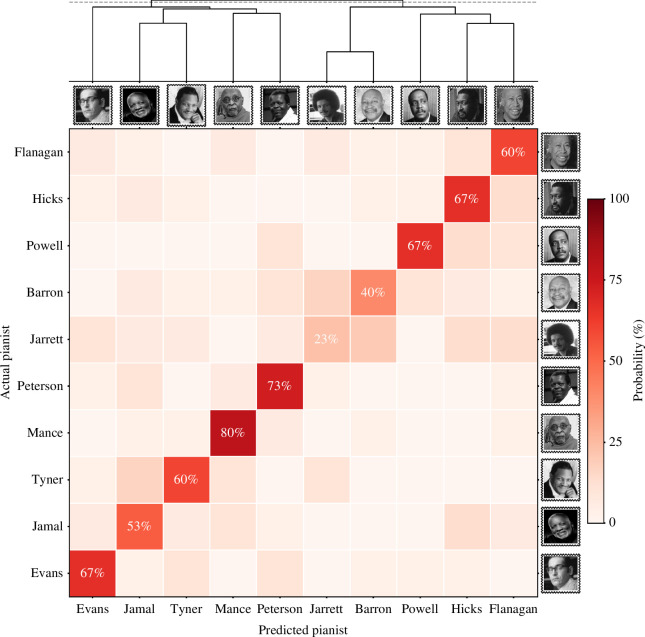
Confusion matrix and clustering dendrogram. The heatmap shows the probability that, when given a recording, the random forest model will identify a particular pianist. The proportion of hits is shown along the diagonal; all other values are misses. Lighter and darker colours indicate lower and higher predictive probability, respectively. The dendrogram above the heatmap shows the results of the hierarchical clustering analysis; pianists joined at lower y-axis values are estimated to be more similar than those joined at higher values. The dashed horizontal line indicates where the dendrogram was cut.

The model was fitted using *k*-fold cross-validation (*k* = 5, 4 : 1 train-test split), stratified such that the balance of tracks by each pianist within the training and testing sets of each fold was maintained (i.e. there were 24 tracks per pianist in every training set). Averaged across all folds, the combined accuracy of the model was 59%, suggesting approximately a three-fifths probability that the pianist in a recording could be identified solely based on the rhythmic qualities of their playing. This was six times higher than chance performance (10%). The accuracy of predictions for individual pianists is shown as a confusion matrix in figure 4. The top-k accuracy (*k* = 3) of the model was 80%—meaning that, for four-fifths of the recordings in JTD-300, the actual pianist was within the three classes with the highest predictive probability estimated by the model.

To better visualize the predictions made by the model, we have created an interactive Web application, accessible at https://huwcheston.github.io/Jazz-Trio-Database/_static/prediction-app.html. This was created by taking the estimated probability of every pianist playing on each track to create a matrix of shape *n*(recordings) × *n*(classes). The dimensionality reduction algorithm t-SNE was then applied to this matrix (with perplexity = 25) to reduce the probabilities for each recording to a single pair of coordinates within a two-dimensional space. The implementation of t-SNE was also from *scikit-learn* [45]. On this Web application, individual points correspond with particular songs in JTD-300 and can be clicked to load the corresponding section of audio within the browser. The confidence of the model predictions are then indicated by the proximity of each point to images of the corresponding pianist.

Both this Web application and figure 4 demonstrate that the model found it substantially easier to classify some pianists than others. It is worth considering why this was the case. Of particular note, here, is that the most accurate classifications were not necessarily obtained for recordings made by the most prolific artists. For instance, Keith Jarrett (accuracy: 23%) has recorded many hours of piano trio material, with his discography the second largest out of all the pianists sampled in JTD, but the model was substantially worse at identifying his recordings than those by any other pianist.

There are several reasons for this. First, the particularities of Jarrett’s style could be primarily harmonic or melodic, rather than rhythmic; without access to this information, the model would have struggled to identify him accurately. Second, the ‘breadth and depth of … influences’ on Jarrett and ‘his ability to blend these various sources of inspiration together’ [46] could have rendered him a stylistic ‘chameleon’, able to imitate the styles of other performers in a way that precludes the easy classification of ‘his’ sound; indeed, the Web application makes it clear how Keith Jarrett’s recordings are frequently mistaken for those by other pianists, especially Kenny Barron. Finally, an audio signal alone might not identifiably reflect the ‘physicality’ and visual aspects of Jarrett’s performance style, which involves idiosyncratic performance mannerisms, vocalizations and an extreme degree of bodily movement [46,47].

### 3.2. ‘Feel’ and ‘complexity’ features best predict identity

**Figure 5.**
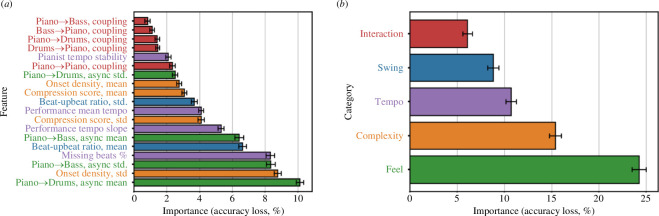
Variable importance scores. (*a*) shows the importance of all features in the random forest. (*b*) shows the importance of all feature categories. The x-axis shows the drop in classification accuracy of the test data when values of the feature or feature group were permuted, averaged over all repetitions of the process. Error bars represent 95% CI.

Next, we turned our attention to the relative importance of the features used by the model. Following Breiman [48], the importance of a feature (or category of features) was computed as the mean decrease in predictive accuracy of the random forest in predictions of the held-out test data within each fold, when values of the given feature (or all the features in a category) were randomly permuted; 200 permutations were completed for each fold and the importance measures were averaged over all repetitions and all folds (i.e. *n* = 1000 total replicates). Feature importance scores are shown for individual features and feature categories in figure 5.

**Figure 6.**
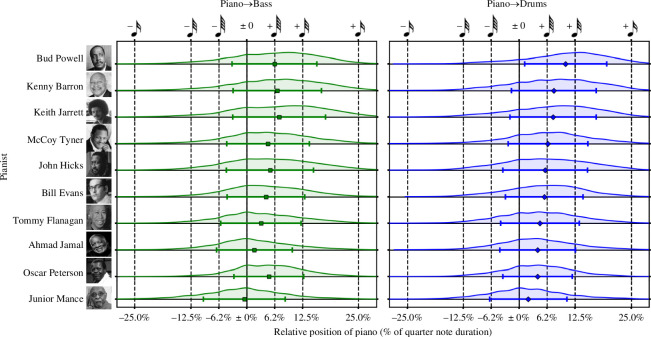
Pianist relative position to accompaniment. The kernel density estimates show the relative position of the pianist to an accompanying instrument (bass, left; drums, right), with positive values indicating that a pianist played after the accompaniment. Markers show mean position, error bars ± 1 SD. Pianists arranged according to mean asynchrony with drums, in descending order.

The single most important feature used by the model was the mean asynchrony between piano and drums; the standard deviation of asynchrony between piano and bass was also the third most important feature and the corresponding ‘feel’ category the most important of all high-level feature categories. Jazz soloists typically mark the musical pulse significantly later than their accompaniment; this phenomenon has been referred to as a ‘relaxed’ or ‘laid back’ feel [17–19] and has previously been demonstrated in JTD recordings [23]. Although the majority of pianists in the dataset typically marked the beat later than both of their accompanists, we still observed substantial variation in rhythmic ‘feel’ between performers (figure 6).

Junior Mance exhibited the closest synchronization with both of his accompanists: expressed as a percentage of the duration of a quarter note, Mance played on average 2.0% (SD = 8.6) behind drummers and 0.5% (SD = 9.1) ahead of bassists. This was somewhat predictable as Mance was the only pianist in JTD-300 to have been active in ‘soul jazz’, a stylistic fusion of jazz, rhythm-and-blues and gospel music that emphasizes rhythmic drive and groove [46]. Here, we note that close—but not completely isochronous—timing between instrumental parts has been noted to enhance subjective perceptions of groove in listeners [40].

Bud Powell, meanwhile, displayed the greatest mean asynchrony with his drummer of any pianist, marking the beat around a 32nd-note later (10.4%, SD = 9.2) and also displayed substantial asynchrony with his bassist, at around a 64th-note lag (mean = 6.2%, SD = 9.5). Powell also displayed the greatest mean tempo instability of all pianists in the database; at 30.4 ms, this was approximately 10% of the duration of a quarter note at the average JTD-300 tempo of 195 BPM.

Although these large asynchronies could have been a conscious decision, biographical evidence may offer another explanation. Following a prolonged series of medical incidents brought on by a racially motivated attack in 1944, Powell was frequently hospitalized during the latter part of his career. It was during this period that ‘his command of the instrument was always more or less impaired’ and he reportedly had to be tranquilized to perform [47]. Most of the recordings in JTD-300 were made during this time, the quality of which have been described by critics as ‘execrable’—‘his touch unsure, the tempo wobbly’ [46]—which could offer one potential explanation for the increased asynchrony in his playing compared with the other pianists in the database.

**Figure 7.**
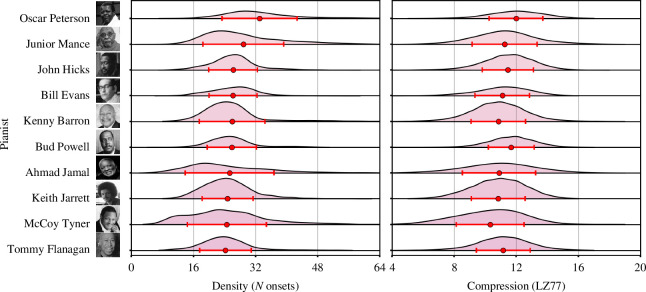
Pianist density and compression scores. Distributions of the density (left) and compression (right) of all onsets within a sliding window of four measures, across all pianists. Markers show mean values, error bars ± 1 SD. Pianists arranged according to mean onset density, in descending order.

The second most important feature used by the model was the standard deviation of windowed onset density measurements, with ‘complexity’ also the second most important category overall. We show kernel density estimates for both compression and density scores across all pianists in figure 7. Ahmad Jamal, a player noted for his use of silence in his improvisations and ‘who combine[d] moments of great power with passages of quiet reflection’ [49], displayed the most variability in the number of onsets played within a typical four-measure span (SD = 11.4, mean = 25). Oscar Peterson’s playing was by far the densest of all pianists in JTD-300, with an average of 33 onsets every four measures (SD = 9.7)—corroborating descriptions of how he produced ‘a multiplicity of notes … in even a relatively subdued context’ [47].

The standard deviation of onset density measurements was approximately three times as important to the model than the mean of these measurements. This might suggest that there was little variation in the average number of notes played by different pianists across four measures, with greater variety to be found in the degree to which they contrasted sparse and dense playing.

Interestingly, the fourth most important feature used by the model—the percentage of tracked beats which could not be matched with a piano onset—followed similar trends to the onset density measure. Oscar Peterson played on the greatest percentage of beats of any pianist, leaving only 17.8% unmarked, while Ahmad Jamal did not play on 31.5% of beats, higher than the average of 28.1%.

**Figure 8.**
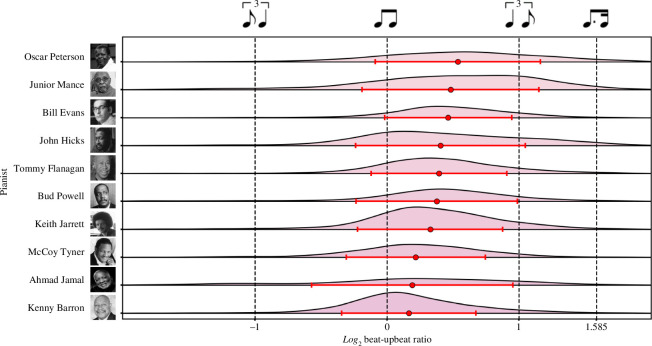
Pianist beat-upbeat ratio distributions. Distribution of log_2_ beat-upbeat ratios obtained for each pianist. Markers show mean log_2_ beat-upbeat ratios, error bars ± 1 SD. Pianists arranged according to mean beat-upbeat ratio, in descending order.

The fifth most important feature used in the model was the pianist’s mean beat-upbeat ratio. While all pianists typically played closer to the equal-equal subdivision of the quarter note (i.e. a 1 : 1 ratio) than the triplet long-short subdivision (2 : 1), we again found substantial variation between performers (figure 8). Oscar Peterson displayed the highest mean log_2_ beat-upbeat ratio (0.84, SD = 0.66), indicative of his ‘unfailing commitment to swinging’ [47], and Junior Mance the second highest (mean = 0.82, SD = 0.69). More surprisingly, however, Bill Evans displayed the third highest mean log_2_ beat-upbeat ratio (0.72, SD = 0.57). Critics of Evans have long focused on a perceived ‘sentimentality’ in his playing, owing to an emphasis on slower-paced ballads and romantic harmony [47,50]. Yet, in his own words, Evans ‘put much more effort, study and development and intensity into just straight ahead jazz playing—swinging, energy, whatever’ [50].

**Figure 9.**
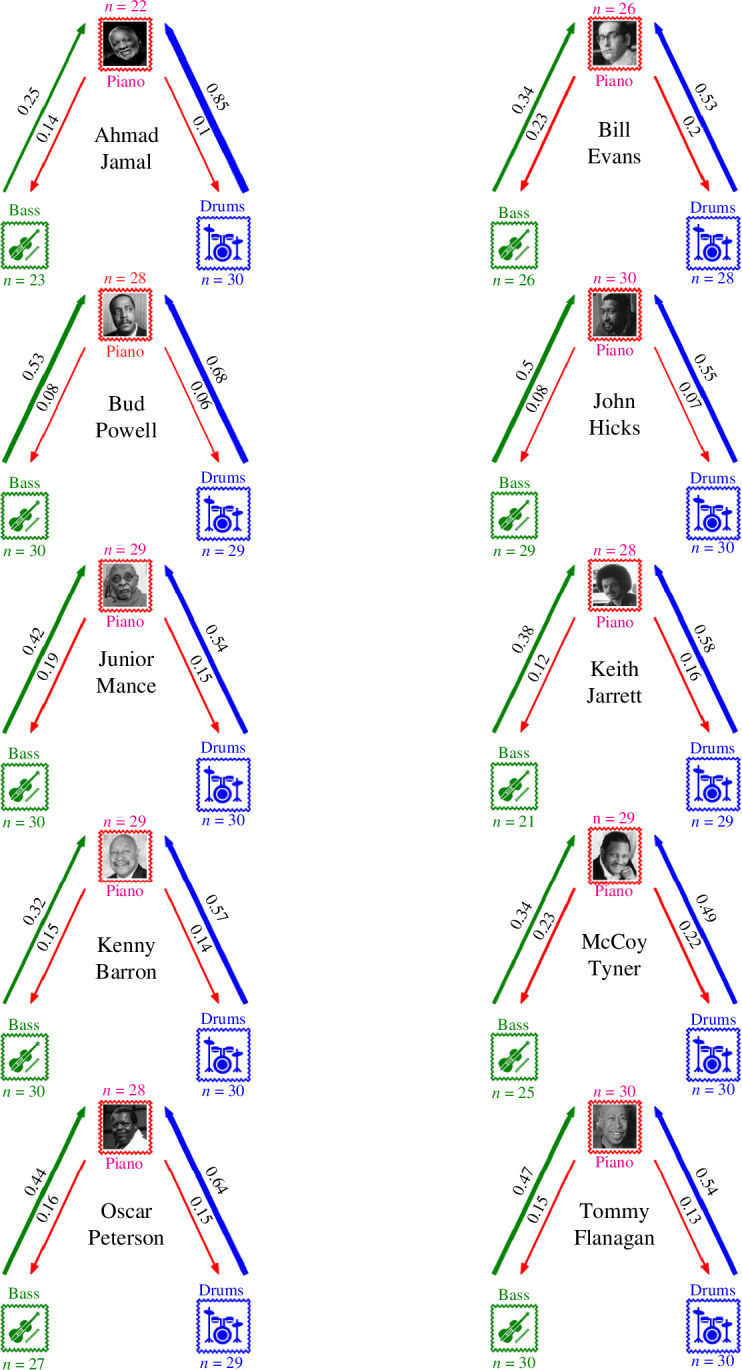
Modelled coupling networks. Individual plots show the average coupling for ensembles led by each pianist. The direction and degree of the coupling is given by the colour and thickness of the arrows, respectively, insofar as an arrow from the pianist to the bassist indicates how much the bassist adjusted to the pianist. Values above each arrow show the mean coupling coefficient. Values of *n* indicate the number of models obtained for that performer or role, with the maximum being 30.

All four of the features with the lowest importance scores belonged to the ‘interaction’ category, which was also the least important feature category overall. While this could be explained by a small number of missing values in this category, which we replaced using global mean scores (see Methods, above), an alternative explanation was simply that the flow of adaptation and coupling was broadly equivalent between ensembles led by different pianists. As shown in figure 9, the drummer exerted the most influence on the pianist in every trio, with the bassist exerting slightly less; additionally, pianists had substantially less influence on these instruments than they had on the pianist. Put differently, ensemble synchronization was typically anchored around the rhythm section of bass and drums, to whom the soloist corrected, rather than the other way around.

This is consistent with previous accounts of musical improvisation in both quantitative and ethnographic research [3,19,22]. For example, Jacoby *et al*. [21] demonstrated that the members of four Malian drumming ensembles adapted most to the performances of the accompanying musicians, rather than to the ‘lead’ drummer. This resulted in lower group asynchrony compared to simulated performances where adaptation was either equally distributed between the performers or anchored entirely around the soloist, whose performance tended to be more variable than the accompanists. This would, in turn, explain the low predictive power of the features from this category: the adaptation between the musicians in each trio functioned primarily as a means for optimizing group synchrony, rather than as a quality manipulated for expressive or aesthetic purposes.

### 3.3. Rhythmic features characterize specific improvisation styles

Next, in order to discern which pianists were considered by the model to be most similar to each other, we performed an agglomerative hierarchical clustering analysis. For each pianist, we created a vector where the *i*th element gave the probability that the algorithm would classify that pianist as pianist *i* (i.e. figure 4, heatmap). We then computed a correlation matrix for these vectors and finally transformed this into a distance matrix by taking 1 – *r* for every element within each vector. The clustering analysis was performed on this distance matrix (using the mean inter-cluster dissimilarity to link clusters together) to create a dendrogram, such that two pianists were considered to be similar if the predictive probabilities estimated by the model for their tracks were positively correlated. The implementation of hierarchical clustering was again from *scikit-learn* [45].

The first split in the dendrogram partitioned the dataset into two clusters, each comprising five pianists: in cluster 1 were Bill Evans, Oscar Peterson, Junior Mance, Ahmad Jamal and McCoy Tyner, while cluster 2 comprised Keith Jarrett, Kenny Barron, Bud Powell, John Hicks and Tommy Flanagan. The two pianists who were considered most similar to each other (i.e. the two to join first in the dendrogram) were Keith Jarrett and Kenny Barron. The clustering analysis also linked several musicians together where biographical evidence suggests that one might have been an influence on the other. Bud Powell, for instance, reportedly influenced a ‘whole school of pianism’, adherents of which included Tommy Flanagan (whose ‘refined keyboard mannerisms’ owed much to Powell) and Keith Jarrett [46]. A further point of comparison was that the median year of all recordings made by cluster 1 pianists was 1969, compared with 1987 in cluster 2—meaning that the latter group may also represent a chronologically ‘later’ style of jazz piano performance.

We show the dendrogram created from this analysis above the heatmap in figure 4; this, in turn, helps clarify how the model often confused pianists from the same cluster for each other, but not pianists from different clusters. In cluster 2, for example, Keith Jarrett was frequently confused for Kenny Barron, but not for any of the pianists in cluster 1. Meanwhile, McCoy Tyner was frequently confused for other cluster 1 pianists, including Ahmad Jamal and Junior Mance, but rarely for any cluster 2 performers. Nonetheless, the heatmap also shows that there is a high degree of heterogeneity within the two clusters on the dendrogram, implying that the individual musicians in JTD-300 still varied substantially in their stylistic signatures.

Next, we wanted to consider the relationship between different aspects of musical rhythm and the makeup of each cluster: whether pianists in one cluster were placed as such because they played with greater density than those in another, for instance. As the directionality of the relationships between feature and target cannot readily be ascertained from a random forest, we performed a *post hoc* analysis using a binary logistic regression model, predicting the cluster assigned to each pianist from the same bag of 19 features used to fit the random forest. The implementation of this model was from the *statsmodels* Python library (v. 0.13.1) [51]. Cluster 2 was used as the outcome (or ‘treatment’) group. Prior to fitting this model, values obtained for each feature were first standardized using *z*-transformation, to enable direct comparison between features on different scales. The total area under the curve between the true- and false-positive rate from this model was 90% (electronic supplementary material, figure S3), substantially greater than chance predictions (50%).

**Figure 10.**
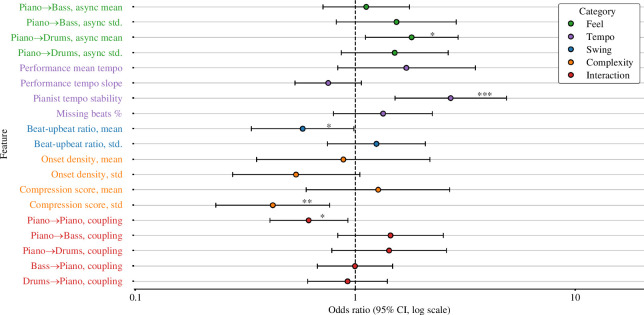
Odds ratios. This forest plot shows results from a binary logistic regression model predicting the cluster assigned to a pianist, with cluster 2 (right-most on dendrogram, figure 4) used as the outcome group. Markers represent the standardized odds ratio (OR) and whiskers 95% CI. A numerical increase in features with OR > 1.0 should be interpreted as higher odds that the pianist came from the outcome cluster 2 (and, vice versa, an increase in features with OR < 1.0 should be interpreted as lower odds). Significant associations are shown with asterisks: ***p < 0.001, **p < 0.01, *p < 0.05.

In figure 10, we plot the odds ratios associated with each feature in the bag. These values can be interpreted as the change in the odds of a pianist placing within the outcome cluster (i.e. the probability of cluster 2 membership divided by the probability of cluster 1 membership) for an increase of one standard deviation in the feature. For features with an odds ratio above 1.0, increases in numerical values obtained for this feature were associated with a greater probability that the pianist would be a member of cluster 2; these features were the mean piano–drum asynchrony (OR = 1.80, 95% CI: [1.11, 2.93]) and tempo instability (2.71, [1.51, 4,85]). Vice versa, for features with an odds ratio below 1.0, increases in numerical values were associated with a lower probability of cluster 2 membership; these included the log_2_ beat-upbeat ratio mean (0.58, [0.34, 0.98]), compression score standard deviation (0.42, [0.23, 0.76]) and piano self-coupling coefficient (0.61, [0.41, 0.92]). In other words, cluster 1 pianists displayed higher levels of swing, greater variation in rhythmic complexity and greater mean synchrony with their accompaniment.

### 3.4. Individual rhythmic styles are consistent over time

Finally, we considered whether the rhythmic style of each pianist changed over the course of their career by leveraging the diachronic construction of JTD-300. If a pianist’s style had changed over time, we assumed that a model trained on their earlier recordings would make inaccurate classifications of their final recordings, and vice versa. To test these hypotheses, we first partitioned the data into two folds, the test set consisting of either the first or last three recordings by each pianist (i.e. 30 recordings total), and the training set every other track (270 recordings, i.e. a 9 : 1 train-test split, c.f. the 4 : 1 ratio used earlier). We then repeated this process 10 000 times but with the partitions chosen randomly and independent of recording date, in order to obtain a permutation-based null distribution for significance testing.

We computed the accuracy of predicting only a pianist’s first three recordings (47%) and only their last recordings (63%); neither was significantly different from the mean accuracy of the null distribution of 56% (permutation test, one-sided, left-tailed *p’*s = 0.17, 0.84, respectively: see electronic supplementary material, figure S4). In other words, as predictive accuracy was not significantly reduced, there was no evidence suggesting that the rhythmic styles of the pianists in JTD-300 had changed over time, at least to the extent that their first or last performances were any harder to classify than all others.

For an alternative test of the possibility that a pianist’s rhythmic style might have changed over time, we fitted linear mixed effects models, using the implementation from *statsmodels* [51]. In each model, the dependent variable was the most important feature from the ‘feel’, ‘complexity’, ‘tempo’ and ‘swing’ categories, as shown in figure 5. The two fixed effects in each model were (1) ‘chronological time’, the recording year of a given track; (2) ‘career progression’, the recording year relative to the pianist’s career trajectory. Values for (1) were scaled such that 0 equalled the earliest year a track in JTD-300 was recorded (1947), and 1 the latest (2015). Values for (2) were scaled such that 0 equalled the year of each pianist’s first recording, and 1 the number of years elapsed from this date, relative to the total duration of Ahmad Jamal’s career—in JTD-300, Jamal was active for the longest length of time of any performer (49 years, from 1955 to 2004). Each model included a random effect of the pianist (both intercepts and slopes).

**Figure 11.**
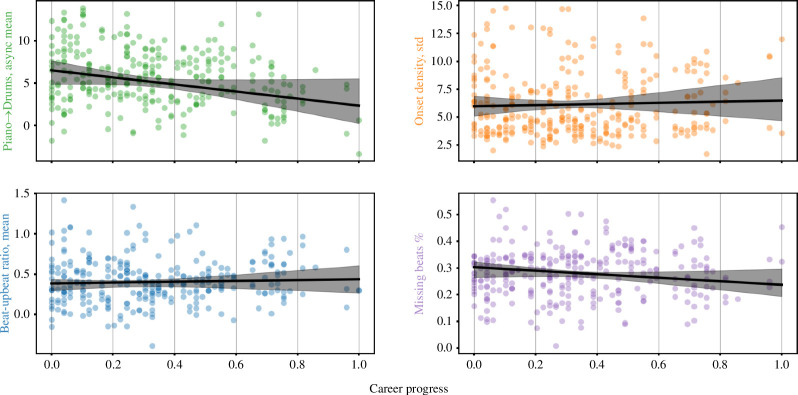
Marginal plots. Each panel shows the relationship between the most important features from the ‘feel’, ‘complexity’, ‘tempo’, and ‘swing’ categories (y-axis) and the ‘career progress’ variable (x-axis). Values of the ‘time’ predictor were set to their mean when predicting y. The random effects included in the models are not shown. Error bars show 95% CI, obtained through bootstrapping (*n* = 10 000 samples).

Neither the ‘chronological time’ nor ‘career progression’ variables predicted significant changes in any of the dependent variables (figure 11). Both fixed and random effects explained an average of 16.9% (SD = 8.93) of the variance in the data across all models, while fixed effects alone only explained 4.50% (SD = 3.71). Again, this reinforced the earlier argument that the rhythmic style of the pianists under investigation remained consistent, both in terms of their individual careers and across the entire span of time covered by the dataset. The greater source of variation was likely to be found instead between different pianists and ensembles, rather than across the output of one individual musician or across different historical periods of jazz. We do, however, note that the recordings in JTD-300 span a relatively narrow range of styles, broadly representative of ‘straight ahead’ jazz, as this has been practised over the last century [23]. One might expect to see such trends across a greater variety of styles, based on the results of e.g. [16].

## 4. Discussion

The purpose of this study was to identify the extent to which performances by an individual musician could be predicted from a supervised learning model trained on a relatively limited set of features—in our case, capturing only the rhythmic qualities of their playing. Nineteen features relating to five categories (‘swing’, ‘complexity’, ‘feel’, ‘interaction’, and ‘tempo’) were extracted automatically from 300 solo improvisations in the JTD by 10 different pianists. These rhythmic features alone were capable of correctly identifying the pianist playing in 59% of recordings, six times better than chance. Important features used by the model in making these judgements related to a performer’s ‘feel’ (their synchronization with the ensemble), ‘complexity’ (the amount of information required to represent the rhythms they played) and ‘swing’ (the subdivision of the pulse into long and short intervals). Further analysis revealed the presence of two clusters of five pianists, with pianists in one category displaying higher levels of swing, greater variation in rhythmic complexity and greater mean synchrony with their accompaniment. Finally, the rhythmic style of individual pianists was shown to change relatively little over the duration of their career. We have developed an interactive web application (huwcheston.github.io/Jazz-Trio-Database/_static/prediction-app.html) that enables the predictions of our model to be explored and the corresponding recordings to be listened to in a browser.

Alongside these analytical insights, we have also demonstrated the applicability of automated methods (supported by recent advances in audio source separation and signal processing) to facilitate musical corpus analyses. Until relatively recently, corpora of recorded music have predominantly been compiled manually, involving lengthy processes of locating audio and annotating it by hand; for examples, see [52,53]. When automatic techniques have been used, this has been restricted to unaccompanied performances by solo instruments, where source separation is unnecessary as a pre-processing step, as in [10,11,13]. Here, we have introduced and verified an automatic method for extracting timing data from group jazz recordings; as the upstream methods used in our analysis pipeline continue to improve, we hope this work will also inspire researchers to apply the procedures used here to a broader range of ensembles and genres.

Our results have implications for the development of music classification systems. When constructing our dataset, we noticed that many of the bassists and drummers in some of the earliest recordings by pianists like Bud Powell and Bill Evans were not initially credited for their performance and so remain unidentified to this day. A similar model to the one constructed here for pianists, but instead trained on the bass and drum data we extracted (and, perhaps, augmented with biogeographical information), could potentially help to identify these players—or, at least, suggest possible stylistic comparisons—many years after the recording was first made. We note that models for attributing authorship both to text and visual art have been built using similar machine-learning techniques to those deployed here [5,6].

In addition, our results have implications for music education. Terms like ‘feel’, ‘swing’ and ‘interaction’ are often used in jazz pedagogy [3], yet defining these with respect to actual performances can be difficult. As part of this project, we have developed a second interactive web application (https://huwcheston.github.io/Jazz-Trio-Database/resources/data-explorer.html) where recordings in JTD-300 can be organized numerically based on the particular features indexed by the model—for instance, showing only those with the strongest sense of ‘swing’ (i.e. highest mean beat-upbeat ratio). Clicking on each track allows it to be listened to directly, with time-synchronized visualizations also included to accompany this analysis. Our hope is that this application offers an intuitive way to highlight particular rhythmic feels—the ‘relaxed soloist’ and so on—by connecting these with recordings that best demonstrate them.

We can foresee several limitations of this work, the first of which concerns the stylistic balance of our dataset. The JTD only contains recordings of ‘straight ahead’ jazz improvisation, made with acoustic instruments and with a clear ‘swing’ rhythmic feel [23]. In using this dataset, we were unable to study important subgenres like jazz fusion, afro-cuban jazz and free jazz, all of which differ from ‘straight ahead’ jazz in their typical use of rhythm [16]. Subsequent projects could use the same methodology we outline here, but apply them to a wider variety of jazz subgenres. Related here is the gender imbalance within JTD-300—although this could be addressed in future work by using the full JTD to train a model, which contains recordings from several important female jazz pianists.

A second limitation relates to our feature extraction procedure. Music is a complex phenomenon, and there is always more detail that could be extracted from an audio recording. To give an example, our approach to modelling rhythmic complexity was relatively simplistic; evaluations of the complexity of a particular span of musical time were only ever made in isolation, without consideration of previous events from earlier in a performance (or, indeed, knowledge of jazz as a whole). Other models of musical complexity make predictions weighted using both the preceding context and training received over a corpus of similar music, meaning that they are thought to better represent an underlying perceptual experience (e.g. [54]). Future analyses of JTD may wish to take just one of these features and explore it in greater detail by applying these models.

A third limitation involved our decision to average several features across an entire performance, which could have smoothed over any meaningful, low-level variation present in (for instance) the extracted beat-upbeat ratio and complexity data [15]. While there is precedent for the use of such ‘global’ features in stylometry [6], future work could instead use features extracted from a smaller section of a recording—predicting the player of a ‘lick’ or phrase, rather than a whole piece. Another possibility would involve training a model to learn meaningful feature representations from an entire performance, capturing patterns and nuances end-to-end from the raw data, without having to aggregate this first.

Finally, it should also be acknowledged here that machine-learning models can learn to focus on features that are highly informative for performer identification and classification, yet relatively imperceptible to listeners. While these models can make highly accurate predictions, it is difficult to claim that they do so using equivalent processes to those involved in human judgements. For instance, there is contradictory evidence as to whether different rhythmic ‘feels’ in jazz can actually be perceived by listeners [17], despite this being among the most important features used by our model. An interesting follow-up project could consider the relative importance of the different features used by human listeners in predictions of musical style and how these might vary depending on levels of experience and knowledge of jazz.

Future work must necessarily expand the definition of musical style to encompass other features beyond rhythm. While it is remarkable that our model could identify the pianist playing in nearly two-thirds of jazz recordings solely by their use of rhythm, this tells only one part of the story: harmony and melody can also be used expressively by pianists, to give but two examples. The JTD also contains pitch and velocity data for pianists, in the form of automatically transcribed MIDI data: a future project could extract equivalent features from these data and combine them with the rhythmic features discussed here to create a larger classification model embodying a broader conceptualization of musical ‘style’.

Taken together, the present study has provided evidence that the stylistic diversity of improvised music stems, at least in part, from rhythmic and temporal variation between different performers. Much work remains before a full understanding of the factors that contribute to distinguishing an individual performer’s ‘sound’ can be ascertained; however, we hope that the public release of the analysis pipeline used in this work will help aid this process.

## Data Availability

Data and relevant code for this research work are stored in GitHub and have been archived within the Zenodo repository: listed in the manuscript references as [55]. The initial paper outlining JTD in detail is also listed in the references as [23]. Supplementary material is available online [56].
